# Acute exposure to a high-fat diet in juvenile male rats disrupts hippocampal-dependent memory and plasticity through glucocorticoids

**DOI:** 10.1038/s41598-019-48800-2

**Published:** 2019-08-22

**Authors:** Tala Khazen, Ossama A. Hatoum, Guillaume Ferreira, Mouna Maroun

**Affiliations:** 1 Sagol Department of Neurobiology, Faculty of Natural Sciences, University of Haifa, Haifa, 3498838 Israel; 2 Department of Surgery B- HaEmek Medical Center, Faculty of Medicine, Technion: Israel Institute of Technology, Haifa, Israel; 3 INRA, Nutrition and Integrative Neurobiology, UMR1286 Bordeaux, France; 4 University of Bordeaux, Nutrition and Integrative Neurobiology, UMR 1286 Bordeaux, France

**Keywords:** Obesity, Long-term memory

## Abstract

The limbic circuit is still undergoing maturation during juvenility and adolescence, explaining why environmental and metabolic challenges during these developmental periods can have specific adverse effects on cognitive functions. We have previously shown that long-term exposure (8–12 weeks) to high-fat diet (HFD) during adolescence (from weaning to adulthood), but not at adulthood, was associated with altered amygdala and hippocampal functions. Moreover, these HFD effects were normalized by treatment with glucocorticoid receptor (GR) antagonists. Here, we examined in male rats whether acute exposure (7–9 days) to HFD during juvenility [from postnatal day (PND) 21 to PND 28–30] or adulthood (from PND 60 to PND 67–69) is sufficient to affect hippocampal functions and whether it is also dependent on GRs activation. Juvenile HFD abolished both hippocampal synaptic plasticity, assessed through *in vivo* long-term potentiation (LTP) in CA1, and long-term hippocampal-dependent memory, using object location memory (OLM). No effect of HFD was observed in short-term OLM suggesting a specific effect on consolidation process. In contrast, adult HFD enhanced *in vivo* LTP and OLM. Systemic application of GR antagonist alleviated HFD-induced LTP and OLM impairments in juveniles. These results suggest that acute exposure to HFD during juvenility is sufficient to impair hippocampal functions in a GR-dependent manner. Interestingly, this effect depends on the developmental period studied as acute exposure to HFD at adulthood did not impair, but rather enhanced, hippocampal functions.

## Introduction

The consumption of a Western diet (containing high levels of fat and sugar) has been shown in both human and animal models to be associated with numerous diseases, including cardiovascular diseases, metabolic disorders, malignancies but also with memory disturbances^1–3^.

In recent years, the hippocampus, a brain structure long considered critical to the performance of a number of learning and memory functions^4,5^, has received increasing attention related to its potential vulnerability to the effects of obesity or obesogenic diet consumption [for review, see^6^]. In particular, rodents that were exposed to several months of high levels of fat or sucrose diets showed impairments in hippocampal-dependent memory^7–10^. Interestingly some studies in adult rodents and humans indicated few days of the obesogenic diet is sufficient to affect hippocampal functioning^11–17^.

The hippocampus is a late maturing structure and undergoes dramatic changes during cortical developments at post-weaning (adolescence in humans). In humans, rapid hippocampal development continues over the first two postnatal years, reflected in gross anatomical changes^18^. Hence, juvenility is considered as critical period in neurodevelopment as changes made during this period have significant effects on lifelong cognitive functions^19^. Recently the effects of high-fat diet (HFD) and/or sugar intake starting at juvenility were examined and showed that the impact of such obesogenic diets for 2–3 months, i.e. covering adolescence, is different from similar exposure but at adulthood. Specifically whereas adolescent exposure was associated with impaired hippocampal functions, similar exposure at adulthood was not associated with any change, suggesting higher vulnerability to the effect of obesogenic diets on hippocampal-dependent plasticity and memory during adolescence^20–25^.

Sparse data addressed the effects of acute consumption of HFD at juvenility without the interference of other maturational processes, such as adolescence that involves sexual maturation. We recently addressed this issue and we showed that 7–9 days of HFD exposure at juvenility is associated with impaired prefrontal plasticity and social memory^26^.

In this study, we sought to compare the effects of short exposure to HFD in the juvenile and adult male rats on hippocampal functions through object location memory (OLM), a task highly dependent on the integrity of the hippocampus (for reviews^27,28^) and *in vivo* hippocampal synaptic plasticity (long-term potentiation, LTP) in the Schaffer collateral-CA1 pathway. As long-lasting exposure to HFD during adolescence was found to involve dysregulation of the glucocorticoid axis^29^, we also addressed the role of glucocorticoids receptors (GR) in acute HFD-induced changes in hippocampal functions. Our results clearly establish qualitative differences between the two age groups and show that acute consumption of HFD impairs hippocampal-dependent memory and plasticity specifically in juvenile animals through GR activation.

## Materials and Methods

### Animals and diets

The experiments were performed using juvenile (28–30 days old) and adult (~70 days old) male Sprague Dawley rats from the local animal colony at the Haifa University. All experimental procedures and protocols were approved by the ethical committee of Haifa University for experimentation with animals and were performed in strict accordance with University of Haifa animal ethical regulations.

The juvenile animals were separated from the dam at the age of 21 days. Maximum of two animals from the same litter were used per experiment. Animals were housed in Plexiglas cages (4–5 rats per cage) and are maintained on a free feeding regimen and a 12 h light/dark cycle. All experiments were performed in the light phase, from 9 am to 1 pm. Juvenile animals were exposed to the regular and standard chow diet, thereafter named control diet (CD) offering 3 kcal/g [consisting of 4% fat and 60% carbohydrate (35% kcal) (ENVIGO, Israel)], or to HFD offering 5.2 kcal/g [consisting of 35% fat, mostly saturated fat from lard (60% kcal), and 26% carbohydrate (20% kcal) (D12492, Research Diets, New Brunswick, NJ)] from post-natal day (PND) 21 to PND 28. Adult animals were exposed to CD or HFD from PND 60 to PND 67–69. Animals were kept on the respective diet until the end of the behavioral or electrophysiological experiments.

### Electrophysiology

Juvenile and adult male Sprague Dawley rats were anaesthetized with a mixture of urethane and chloral hydrate [40% urethane, 5% chloral hydrate in saline; 0.5 ml/100 g, i.p. and supplementary injections (0.1–0.2 ml) were given when necessary to ensure full anesthesia] and placed in a stereotaxic frame (Stoelting, USA). Body temperature maintained at 37 ± 0.5 °C. Small holes were drilled into the skull to allow the insertion of electrodes into the brain.

A bipolar 125 µm stimulating electrode was inserted into the rat Schaffer collateral (Juvenile: −3 mm posterior, 0.2 mm lateral, 3.2 mm ventral to bregma; Adults: −3.1 mm posterior, 0.3–0.5 mm lateral, 3.5 mm ventral to bregma^30^.

A single recording microelectrode (glass, tip diameter 2–5 mm, filled with 2 M NaCl, resistance 1–4 MO) was slowly lowered into the CA1(Juvenile −4.1 mm anterior, 2.1 mm lateral, and 2.8 mm ventral to bregma; Adults: −4.2 mm anterior, 2.5 mm lateral, and 3 mm ventral to bregma). The evoked responses were digitized (10 kHz) and analyzed using a Cambridge Electronic Design 1401-plus data acquisition system (Cambridge, UK) and its Spike2 software. Offline measurements were made of the amplitude of field post-synaptic potentials (fPSPs) using averages of five successive responses to a given stimulation intensity applied at 0.1 Hz. Test stimuli (monopolar pulses; 100 ms duration) were delivered at 0.1 Hz. After positioning the electrodes, the rats were left for 30 min before commencing the experiment.

### Long-term potentiation (LTP)

LTP was induced using the Theta Burst Stimulation (TBS) protocol, which was applied in all groups within 1.5 h of anesthesia. Theta-like high-frequency stimulation was delivered at Schaffer Collateral [TBS: three sets of 10 trains, each train consisting of 10 pulses at 200 Hz for juvenile animals and 100 Hz for adult animals; intertrain interval: 200 msec; 8nterest interval: 1 min]. We used two different protocols of TBS in order to induce similar and comparable levels of potentiation in both ages^31^.

TBS was delivered at the same intensity and pulse duration as the test stimuli during establishment of the baseline responses. Evoked fPSPs at the baseline intensity were recorded from the CA1 for up to 60 min following the application of TBS. The results are presented as the mean of each 5 min period to give 12 time points. LTP was defined as an increase in the amplitude of the fPSPs. Changes in fPSP amplitude were measured for each rat as a percentage of change from its baseline.

### **Object location memory (OLM)**

Animals were tested in a black open field (open field (50 × 50 × 50 cm) under dim light. Animals were habituated to the arena for 10 minutes for two days before the training session. In the training session (day1), rats were first placed individually in the center of the open field. Two different objects were located in opposite corners; each rat was allowed to explore the objects (A and B) for 5 min unless otherwise indicated. The test phase was given 3 h (short-term memory test) or 24 h (long-term memory test; day 2) after the training session. During the 5-min test trial, the rat was presented with the same two objects one of the objects was moved to a new location and the time spent exploring the objects at the old and the new location was recorded for 5 min. The open field and the objects were thoroughly cleaned between trials with 70% alcohol clean wipes.

Exploration was defined as when the subject sniffed at, whisked at, or looked at the object from no more than 2 cm away. A discrimination index calculated for each animal was expressed as *T*N/(*T*N + *T*F) *T*N = time spent exploring the object in the novel location; *T*F = time spent exploring the object in the familiar location). Intact recognition memory in the test phase is reflected in a discrimination score higher than 0.5, which implies greater exploration of the object in the novel location.

### Elevated plus maze test

The elevated plus maze was placed 70 cm above the floor and consisted of two open arms and two closed arms (with 30 cm high walls and no roof), arranged in a way that similar arms are opposite to each other. Following 5 min of habituation to the room, the animal was placed in the center of the maze, facing an open arm, and allowed to explore the arena for 5 min, while its behavior was videotaped. We recorder the distance traveled and the time spent in open and closed arms. An anxiety index was calculated for each animal and was expressed as *T*O/(*T*O + *T*C) [*T*O = time spent in the open arms; *T*C = time spent in the close arms].

### Drugs

The GR antagonist RU486 (RU; Sigma) was first dissolved in 100% ethanol and subsequently diluted in saline to reach the appropriate concentration of 10 mg/kg [based on previous results^32^]. The final concentration of ethanol was 1%. Controls were given the vehicle (1% ethanol) only. Unless otherwise indicated, RU/vehicle was injected i.p. immediately after the sample phase of OLM in order to avoid any potential confounding effect on the performance/acquisition during the training session[for example see^33^]. For electrophysiology RU/vehicle was injected 30 minutes before HFS^29^.

### Plasma corticosterone levels

Corticosterone was measured from trunk blood samples coming either from home cage animals (Basal condition) or 45 min after exposing rats for 10 min in a new open-field, in a new room (New condition). In each condition, rats were either juveniles or adult exposed to CD or HFD.

The total corticosterone level was measured, in plasma obtained after centrifugation of the blood samples (10,000 rpm, 10 min, 4 °C) using MILLIPLEX MAP Rat Stress Hormone Magnetic Bead Panel Kit (#RSHMAG-69K, Milliplex, Germany).

### Western blot

Rats were sacrificed and brain tissues of the CA1 were collected and homogenized in buffer. The samples were diluted in SDS sample buffer, boiled (100 °C) for 5 min and stored at 80 °C. Equal total amount of protein was loaded in each lane (10 μg) of SDS-PAGE gels (10% polyacrylamide).

After standard electrophoresis the proteins were transferred to a nitrocellulose membrane (0.45 μm; Wotman) and the bands were visualized using Ponceau staining (Bio-Rad). Membranes were blocked for 1 h at room temperature with blocking buffer [3% blotting milk in TBS containing 0.1% Tween 20 (TBS-T)]. Membranes were incubated with the GR antibody/Actin (Cell Signaling technology overnight at 4 °C (1:100, Pierce Antibodies) followed by washing and 1 h incubation with corresponding secondary antibody (Cell Signaling technology) at room temperature.

Proteins were visualized by enhanced chemiluminescense (ECL Western blotting analysis system; GE Healthcare) and quantified with a charge-coupled device camera (XRS; Bio-Rad). Each protein sample was measured relatively to the background. To confirm equal protein loading, the same blots were re-hybridized with antibodies specific for β-actin (1:10000, chicken antibody; Abcam, ab13822). No significant difference in β-actin levels was observed between the groups, suggesting that β-actin levels were not affected by the treatment. All protein samples were therefore standardized with β-actin.

### Statistical analysis

Data were expressed as the mean ± SEM, and statistical analyses were conducted using SPSS (version 21) or Prism 7 software with a threshold for considering statistically significant difference being set up at *p* ≤ 0.05. Body weight data were analyzed using unpaired Student’s *t* tests. The OLM, LTP and corticosterone results were analyzed using ANOVAs with diet (CD vs HFD), age (juvenile vs adult) or drug treatment (RU486 vs vehicle) as between-subject factors and with repeated measurement on the time factor when appropriate. This was followed by simple effects analyses to establish the source of any significant interactions. Analysis of Western blot data was performed using Quantity One computer software. Statistical analysis was performed using independent sample *t* tests (*t* tests were performed only on data from the same gel).

## Results

### The effects of HFD exposure on bodyweight

We monitored the consumption of HFD on bodyweight change and did not find any significant differences between the animals that were exposed to HFD as juveniles (jHFD) or adults (aHFD) and their corresponding controls exposed to the regular diet [jCD or aCD; F(1, 22) < 1 for the effect of Diet; Table 1, also see^26^].

**Table 1:** Juvenile and adult HFD fed animals do not show an increase in body weight following 7 days of HFD: In a sample of animals, we monitored the body weight before and after the 7 days exposure to CD or HFD.

	jCD (n = 11)	jHFD (n = 10)	aCD (n = 10)	aHFD (n = 10)
Initial body weight (g)	46 ± 2.4	43 ± 3.7	304 ± 13.4	309 ± 9.1
Final body weight (g)	98 ± 1.7	107 ± 1.9	343 ± 13.3	354 ± 13.1

Only Age effect was observed [Age: F(1, 22) = 425.5, P < 0.001, without an effect of Diet: F(1, 22) = 1.3, or of interaction Age × Diet: F(1, 22) = 1.4].

### Opposite effects of acute exposure to HFD on hippocampal-dependent memory and plasticity in juveniles and adults

**Figure 1:**
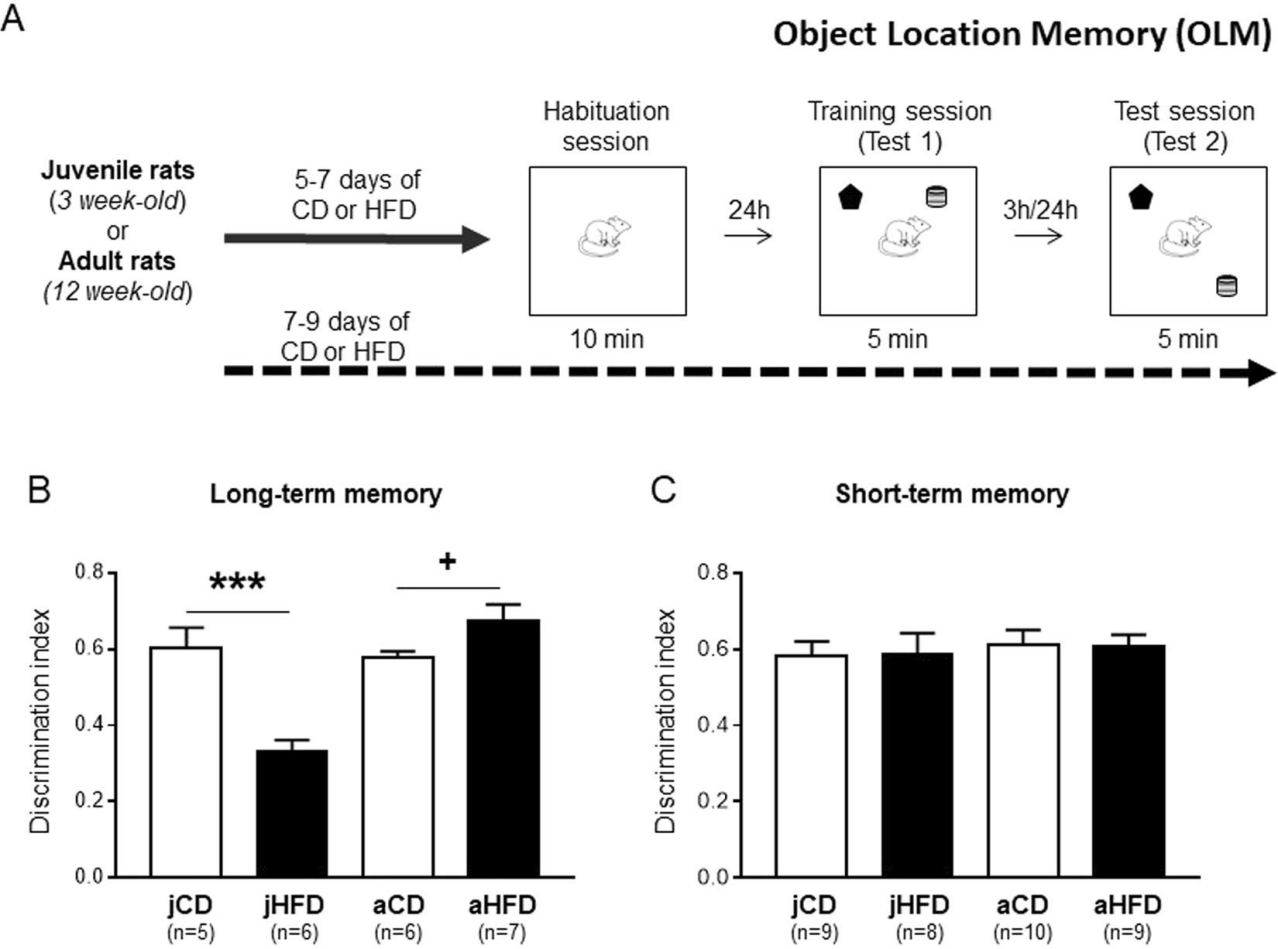
Opposite effects of acute exposure (7 days) to HFD on object location memory (OLM) in juvenile and adult male rats (**A**) Schematic representation of the experimental procedure. (**B**) A significant interaction between diet and age of exposure was observed in the long-term OLM [F(1, 20) = 23.7, P < 0.001]. Whereas acute exposure to HFD impaired long-term OLM in juvenile rats (***P = 0.001), HFD enhanced OLM in adults (^+^P = 0.075). (**C**) No significant effects were observed between the groups in the short-term OLM [F(1, 32) = 1.4].

We first assessed whether 7 days exposure to HFD after weaning or at adulthood would affect short- and long-term object location memory (OLM). Schematic presentation of the procedure is described in Fig. 1A. The groups were as following: Long-term memory [jCD (n = 5), jHFD (n = 6); aCD (n = 6), aHFD (n = 7)], Short-term memory [jCD (n = 9), jHFD (n = 8); aCD (n = 10), aHFD (n = 8)].

The groups did not differ in the exploration ratio during the training session [Age (juveniles vs. adults) × Diet (CD vs. HFD); no effects of Age, Diet or interaction: F < 1]. When long-term memory was assessed 24 hours after training, ANOVA revealed significant effects of Age [F(1, 20) = 17.5, P < 0.001], Diet [F(1, 20) = 5.4, P < 0.05] and interaction between Age and Diet [F(1, 20) = 23.7, P < 0.001; Fig. 1B]. Post-hoc analysis showed opposite effects for the HFD compared to CD in juveniles and adults. In juveniles, HFD significantly impaired long-term memory (P = 0.001; Fig. 1B) whereas in adults, HFD was associated with a trend to enhance long-term memory (P = 0.075; Fig. 1B).

When short-term memory was assessed 3 hours after training, the groups did not differ [Age, Diet or interaction: F(1, 31) < 1; Fig. 1C] suggesting a specific HFD effect on long-term memory consolidation for both juveniles and adults.

It should be noted that these HFD effects on long-term OLM could not be attributed to differences in exploration time as the groups did not show any significant differences on exploration time of the two objects during the training or the test session (See Supplementary Table 1).

**Figure 2:**
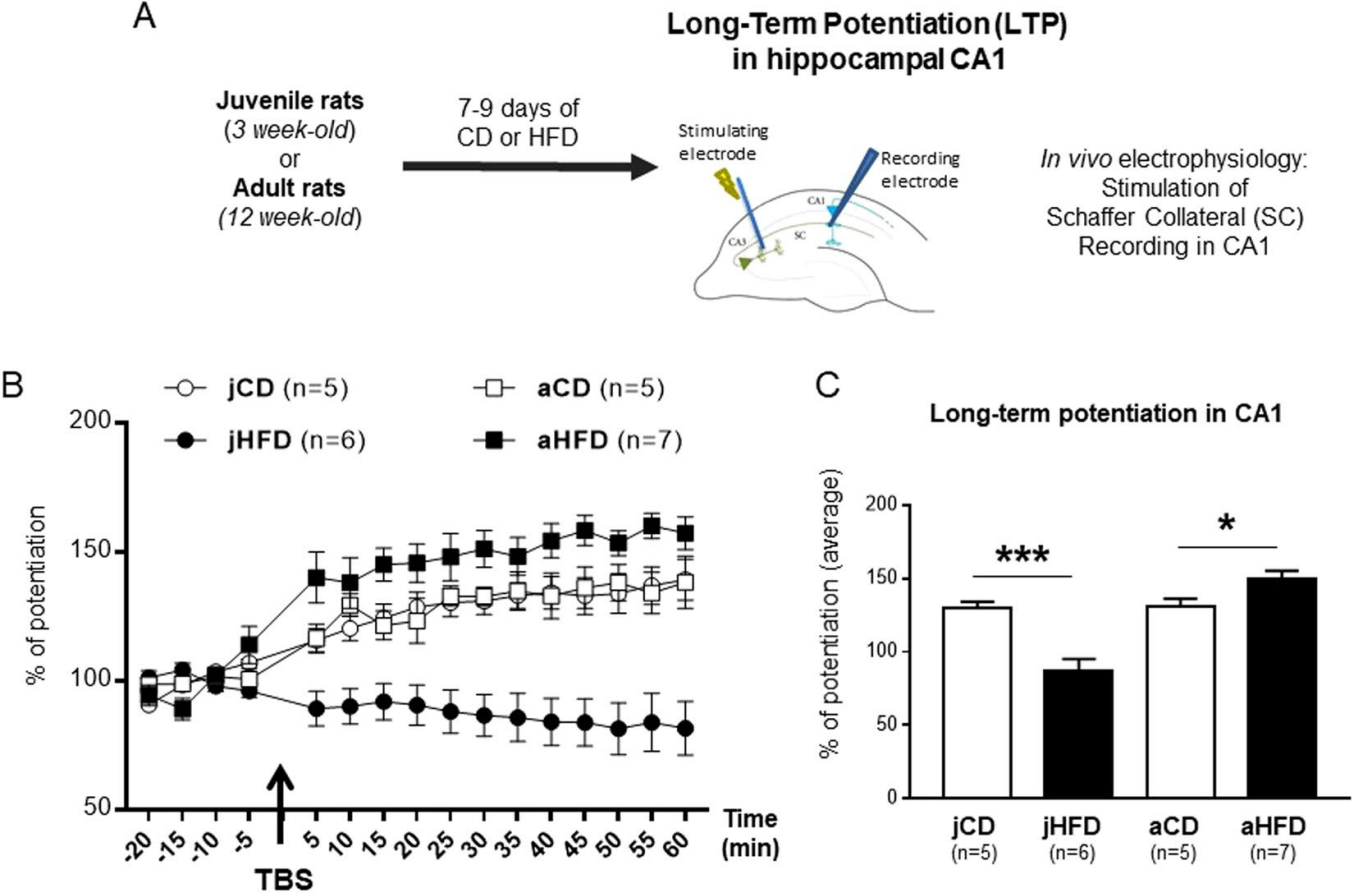
Opposite effects of acute exposure (7 days) to HFD on hippocampal plasticity in juvenile and adult male rats. (**A**) Schematic representation of the experimental procedure. (**B**) Time course plots of normalized field post-synaptic potentials (fPSPs) recorded *in vivo* before and after theta burst stimulation (TBS) of the Schaffer collateral-CA1 pathway. (**C**) Bar histogram representing percentage of long-term potentiation (LTP) during the 40 min after TBS in the same groups as described in (**B**). A significant interaction between diet and age of exposure was observed [F(1, 19) = 24.8, P < 0.001] with exposure to HFD impairing LTP in juvenile animals (***P = 0.002) whereas in adults, HFD exposure enhanced LTP (*P = 0.04).

The behavioral results motivated us to ask whether hippocampal plasticity will be affected in juvenile and adult animals following acute (7–9 days) exposure to HFD. We thus assessed whether similar period of exposure to HFD after weaning or at adulthood would also affect *in vivo* CA1 LTP (Fig. 2A). The groups were as following: [jCD (n = 5), jHFD (n = 6); aCD (n = 5), aHFD(n = 7)]. No differences between the different ages or following HFD intake were observed in the baseline amplitude [F(1, 19) < 1] or in the stimulation intensity required to produce comparable baseline signal [F(1, 19) < 1]. After stimulation (TBS), ANOVA [Age (juveniles, adults) × Diet (CD, HFD)] revealed significant interaction between Age and Diet on the average LTP during the 40 min after TBS [F(1, 19) = 24.8, P < 0.001; Fig. 2B,C]. Post-hoc analysis showed opposite effects of HFD in adults and juveniles. In juveniles, HFD was associated with impaired LTP (P = 0.002) whereas in adults, HFD exposure was associated with enhanced LTP (P = 0.04; Fig. 2C).

Altogether, these results point that short exposure to HFD results in opposite effects on hippocampal-dependent memory and plasticity in juveniles and adults, resulting in impaired hippocampal functions in juveniles but improved functions in adults.

### Acute exposure to HFD alters novelty-induced glucocorticoids release in juveniles but has no effect on glucocorticoid receptor expression in hippocampus

**Figure 3:**
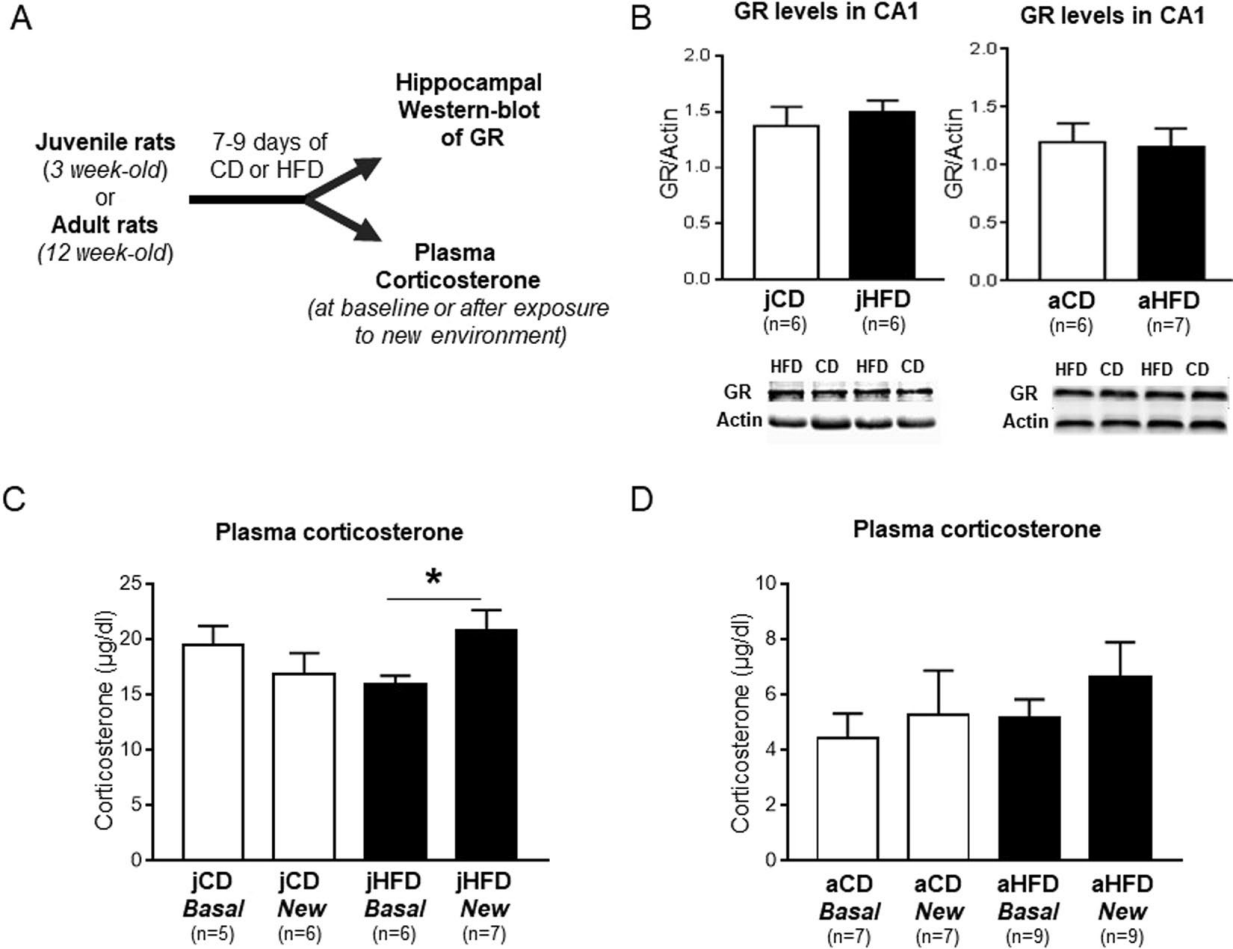
Effects of acute exposure (7 days) to HFD on glucocorticoid receptor (GR) expression in hippocampus and novelty-induced plasma corticosterone release in juvenile and adult rats. (**A**) Schematic representation of the experimental procedure. (**B**) Acute exposure to HFD did not affect the levels of GR expression in CA1 in either juvenile [t(10) < 1] or adult rats [t(11) < 1]. (**C**) Plasma corticosterone increased after exposure to new object in a new open field (New) in HFD-fed juvenile animals [F(1, 20) = 4.9; *P < 0.05] but not in CD group [F(1, 14) = 1.1, n.s]. (**D**) No significant differences in levels of plasma corticosterone were observed in adult animals [F(1, 28) = 0.70, n.s].

As we recently showed that the memory and plasticity alterations induced by chronic exposure to HFD at juvenility involved dysregulation of the glucocorticoid axis^29^, we evaluated whether this dysregulation is present after acute HFD. We first examined whether acute (7–9 days) exposure to HFD affected the hippocampal levels of glucocorticoid receptors (GR) expression in CA1 using Western blots. The groups were as following: [jCD (n = 6), jHFD (n = 6); aCD (n = 6), aHFD(n = 7)]. There were no significant differences in GR expression in CA1 compared between CD and HFD groups in either juveniles or adult rats [t(10) < 1 and t(11) < 1, respectively; Fig. 3B, full-length gels are presented in Supplementary Figs 1 and 2].

We then evaluated whether acute (7–9 days) exposure to HFD could affect plasma corticosterone (cort) levels measured at baseline (*Basal* condition) or after exposure to novel environment in an open field (*New* condition), used as mild psychogenic stressor (see for instance^34^). The groups were as following: [jCD Basal (n = 5), jCD New (n = 6), jHFD Basal (n = 6), jHFD New (n = 7); aCD Basal (n = 7), aCD New (n = 7), aHFD Basal (n = 9), aHFD New (n = 9)]. In juveniles, the results show significant interaction between Diet and Condition [F(1, 20) = 4.9; P < 0.05; Fig. 3C]. Follow-up analysis showed that jHFD and jCD groups did not differ in the baseline levels of plasma cort (P > 0.1). However, following exposure to a new open-field, HFD-fed juveniles showed an increase in plasma cort (jHFD-New *versus* jHFD-Basal; P = 0.045) whereas no increase was apparent in CD rats (jCD-New *versus* jCD-Basal; P > 0.1; Fig. 3C). In adults, no significant interaction between Diet and Condition was observed [F(1, 28) < 1; Fig. 3D].

### Glucocorticoid receptors blockade rescued HFD-induced memory and plasticity impairments in juvenile animals

**Figure 4:**
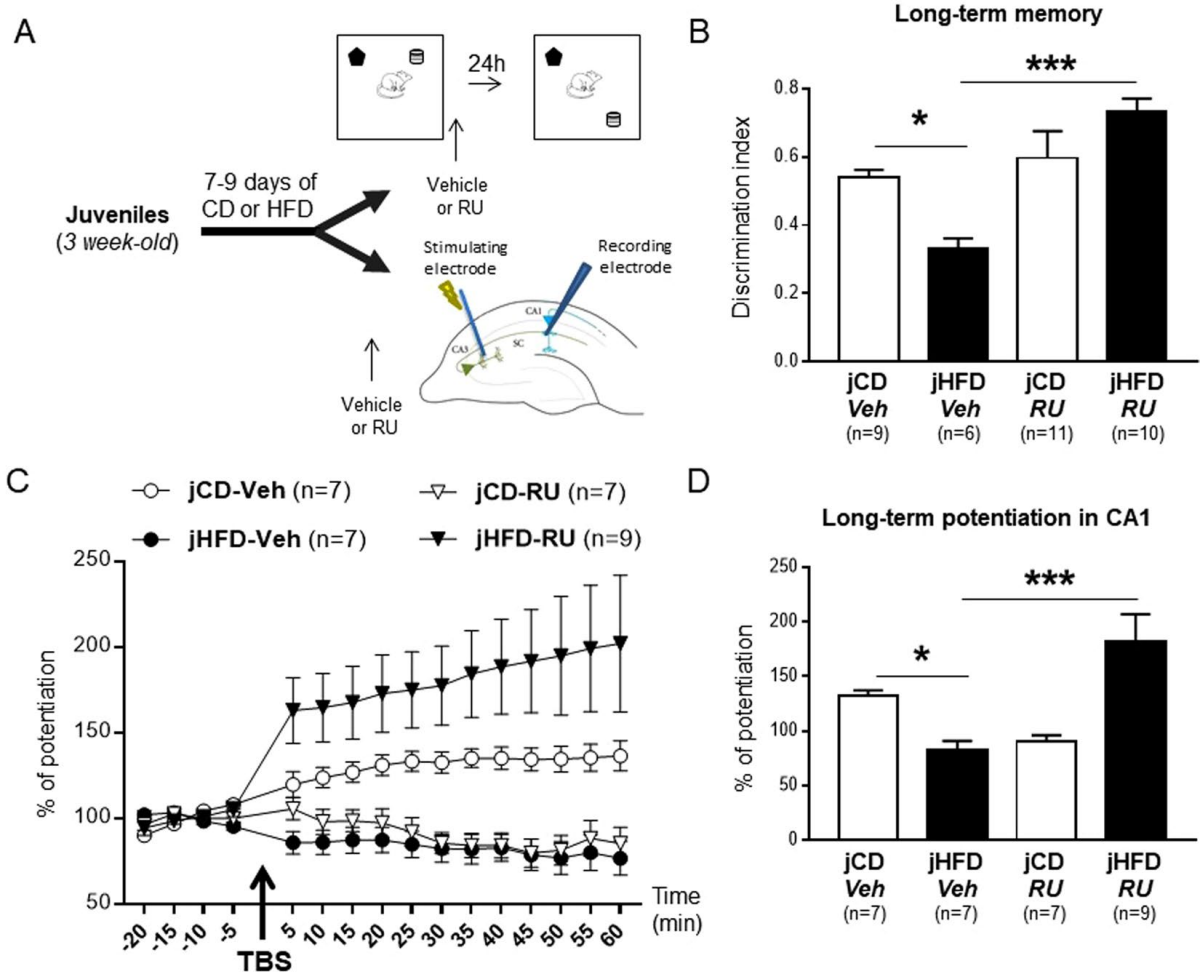
Effects of glucocorticoid receptors blockade on juvenile HFD-induced impairments of object location memory and hippocampal plasticity. (**A**) Schematic representation of the experimental procedure. (**B**) HFD impaired long-term OLM in vehicle (Veh)-treated juveniles, and GR blockade with RU486 (RU) rescued OLM in the HFD group without affecting OLM in CD groups [Interaction Drug × Diet: F(1, 32) = 9.15 ***P = 0.005]. (**C**) Time course plots of normalized field post-synaptic potentials (fPSPs) recorded *in vivo* before and after theta burst stimulation (TBS) of the Schaffer collateral-CA1 pathway. (**D**) Bar histogram representing percentage of long-term potentiation (LTP) during the 40 min after TBS in the same groups as described in (**B**). HFD impaired LTP in Veh-treated juveniles, whereas prior with RU treatment in HFD fed animals restored LTP [t(14) = 3.6, ***P = 0.005]. Similarly, RU also impaired the induction of LTP in CD animals [t(2) = 5.0, P = 0.001].

As only the HFD-fed juveniles showed changes in plasma cort levels following exposure to the new open field and plasticity and memory impairments, we focused on juveniles and evaluated whether blocking GR could improve the effects of acute jHFD on OLM and LTP. We first addressed the effect of the GR antagonist RU486 on OLM by injecting the drug immediately after the training session (Fig. 4A). The groups were as following: [jCD-Veh (n = 9), jHFD-Veh (n = 6); jCD-RU (n = 11), jHFD-RU (n = 10)]. No significant differences between the groups was found in the exploration ratio during the training session [Drug × Diet interaction: F(1, 32) < 1; see Supplementary Table 1 for exploration time]. In contrast, during the test session, significant effects of Drug [F(1, 32) = 16.1, P < 0.001] and interaction between Drug and Diet [F(1, 32) = 9.15, P = 0.005] were found (Fig. 4B; Supplementary Table 1 for exploration time). Post-hoc analyses indicated that, as expected, HFD disrupted long-term OLM as compared to CD in vehicle-treated juveniles (P < 0.05) and that GR antagonist enhanced OLM in HFD-fed juveniles (P < 0.001) whereas it did not affect performance in CD groups (P > 0.1). Surprisingly, the jHFD-RU animals showed even higher performance than the jCD-Veh animals, suggesting enhanced memory (P < 0.05).

In order to confirm that these effects are specific to the consolidation phase, we evaluated in CD and HFD-fed juveniles the effect of GR blockade 6 hours after the training session (i.e. out of the window of consolidation) on long-term OLM tested the next day (Supplementary Fig. 3A). No significant differences between the groups was found in the exploration ratio during the training session [Drug × Diet interaction: F(1, 18) < 1; Supplementary Table 1]. During the test session, there was a strong effect of Diet [F(1, 18) = 85.1, P < 0.0001] without interaction between Drug and Diet [F(1, 18) < 1] indicating that juvenile HFD induced a strong OLM impairment that was not rescued by the GR antagonist (Supplementary Table 1 and Fig. 3B). Thus, these results suggest that the effect of RU486 on reversing jHFD-induced OLM impairment is specific to the consolidation window.

To rule out that the beneficial effect of GR antagonist on long-term OLM was due to changes on basal locomotor activity or anxiety levels, we examined the effect of juvenile HFD and RU treatment on elevated-plus-maze (EPM) task conducted 24 hours after systemic injection of RU or Vehicle. Two-way ANOVA revealed no significant effect of Diet and drug interaction on total distance travelled in EPM [F(1, 15) = 3.4, P = 0.09] and on the anxiety index [F(1, 15) < 1; Supplementary Fig. 4A,B]. Together, these results confirm that the long-term OLM improvement of HFD-fed juveniles treated with RU is not due to changes in locomotor activity or anxiety levels.

We finally evaluated the effect of the GR antagonist on CA1 LTP (Fig. 4A). The groups were as following: [jCD-Veh (n = 7), jHFD-Veh (n = 7); jCD-RU (n = 7), jHFD-RU (n = 9)]. No effect of HFD or Drug were observed in the baseline amplitude or in the stimulation intensity required to produce comparable baseline signal (F < 1). Repeated measures ANOVA on post HFS time points revealed a significant interaction of Diet and Drug [F(1, 26) = 18.8, P < 0.001; Fig. 4C]. Post-hoc analysis showed that, as expected, HFD impaired LTP in vehicle-treated juveniles (P < 0.05 for average LTP; Fig. 2C), whereas RU486 treatment restored LTP in HFD fed animals (jHFD-RU *versus* jHFD-Veh; P < 0.001 for average LTP), it blocked LTP induction in CD (jCD-RU*versus* jCD-Veh; P = 0.005 for average LTP). Further, jHFD-RU group showed higher LTP than the jCD-Veh and jCD-RU animals (P > 0.05 and P < 0.001 for average LTP, respectively). Thus, GR antagonist rescues impairments of both long-term OLM and CA1 LTP induced by acute exposure to HFD in juvenile animals. This indicates that GR over-activation mediates juvenile HFD-induced impairments of hippocampal-dependent memory and synaptic plasticity.

## Discussion

Using behavioral and electrophysiological approaches and comparing between the juvenile and adult male rats, we report three major findings: (1) 7–9 days of HFD exposure is sufficient to induce changes in hippocampal-dependent long-term memory and hippocampal CA1 LTP in both juvenile and adult animals, (2) the response profile of juveniles is opposite to that of the adult animals and (3) HFD-induced memory and LTP impairments in juveniles depend on GRs activation.

While several previous studies show the impact of chronic consumption of HFD (from 8 to 16 weeks) during adolescence on the physiology of the brain, especially of the hippocampus^20–22,25,35^ none examined the acute impact of HFD on hippocampal functions in the juvenile animal. We investigated the effects of HFD consumption for 7–9 days and we demonstrate that juvenile consumption of HFD impairs long-term memory of object location as well as the induction of *in vivo* LTP in the CA1. In contrast, similar acute HFD exposure in the adult animal slightly enhances *in vivo* LTP and OLM. Interestingly, no effect of HFD was observed 3 hours following the training phase as no differences were reported between CD and HFD animals. This finding suggest (1) the changes in long-term memory could not be attributed to general impairments in sensory, perception or motor functions and (2) HFD specifically affected memory consolidation as previously reported for longer HFD exposure during adolescence^20,29^ and after acute exposure (3 days) to HFD in aged rats of 24 months^17^. Interestingly, this study also indicated that acute HFD exposure did not affect short and long-term memory in adult male rats (3 months old^17^) suggesting that adolescence and ageing could represent particular and similar vulnerable periods to the neurocognitive effects of HFD. Further, the differential effects of HFD on OLM in adults and juveniles could not be attributed to differences in exploration time as both ages showed similar exploration during the sample and testing phases.

Our data points that, for either juveniles or adults, HFD induces similar effects on long-term memory and LTP suggesting a possible link between the two phenomena in the effects of HFD on these processes, in a similar way to the effect that was reported for stress for example (e.g^36^). These data also demonstrate, in confirmation of our recent findings, developmental differences following different manipulations (exposure to either diet or behavioral stress) in the juveniles as compared to the adult animals^20,29,31,35,37^. Regarding plasticity, we recently reported that juvenile animals differ in the ability to induce LTP in the medial prefrontal cortex compared to adults^37^ and that different protocols are needed for the induction of LTP in juveniles as compared to the adults^26^. Here again, different protocols were used to obtain similar levels of potentiation, stressing again developmental peculiarities. This could be attributed to the ongoing maturation of the hippocampus and the prefrontal cortex in the juvenile animals that may set a higher threshold for the induction of LTP.

The enhanced LTP and memory in the adult HFD-fed animals may be consistent with the recent findings that 3 days of HFD intake in adults resulted in increased hippocampal levels of galanin and brain-derived neurotrophic factor (BDNF)^38^, which are markers for neuroprotection and plasticity, respectively. Future studies should determine whether this up-regulation of galanin and BDNF in hippocampus could be the substrate that leads to enhancement of hippocampal plasticity and memory in adult animals following brief exposure to HFD. Interestingly, the present results and those of Gan and colleagues^38^ are in contrast to previous findings indicating that chronic exposure to HFD at adulthood was not associated with any change in hippocampal function^20^ or impairs this function^7–10^. Overall, these results indicate that in adult animals the acute response to HFD is entirely different from a chronic exposure and suggests that HFD exposure at adulthood may be progressively associated with adaptive changes as recently shown in adult HFD-fed rats^15^.

## Role of the Glucocorticoids Receptors

We previously reported that the effects of exposure to HFD starting from juvenility depend on the glucocorticoid axis. We specifically found that juvenile animals fed for 3 months on HFD showed enhanced cort levels following stressful events (psychogenic or systemic stressors) as compared to the control-fed animals. Yet, no differences in baseline levels of plasma cort were observed^29^. In the present study, we chose to use a mild stressor to prevent a ceiling effect and to be able to detect possible differences between the different groups. We show that mild psychogenic stressor, i.e. exposure to novel environment, is associated with a slight increase in plasma cort only in the juveniles-HFD animals. This may suggest juvenile HFD consumption decreases the threshold for the activation of the glucocorticoid axis as the other groups did not show an increase in cort levels following the exposure to the novel environment. The results also point that even in the baseline there are differences in the levels of plasma cort between adults and juveniles. We have previously showed similar results prior to the exposure to the elevated platform stressor^37^. Specifically, the juvenile animals showed higher baseline levels compared to the adults; however, exposure to stress similarly increased the plasma corticosterone. Further, differences were reported between adults and juveniles in the hypothalamic-pituitary-adrenal axis response^39,40^. Additionally, the regulation of the hypothalamic-pituitary-adrenal axis undergoes extensive morphologic and functional remodeling during this period^19,41–43^.

Based on these findings, we proceeded to address whether systemic blockade of the GR is associated with any change of the HFD-induced effects on hippocampal function, i.e. memory and synaptic plasticity. We found that systemic blockade of GR restores LTP in Schaffer Collateral-CA1 pathway and long-term OLM of juvenile HFD-fed rats, suggesting that HFD resulted in activation of the hypothalamic-pituitary-adrenal axis in juvenile rats. Interestingly, monitoring GR expression in the CA1, we did not observe difference between the groups suggesting that the effects of juvenile HFD is not due to enhanced GR expression in the hippocampus but rather GR over-activation. Interestingly, we showed that the reversal of memory impairments following RU486 in HFD is not due to changes in locomotor activity or in anxiety levels. Further, RU486 is effective in reversal of HFD effects on OLM only when it is administered immediately after training indicating a specific beneficial effect on the consolidation process. As we used systemic injections of GR antagonist, we cannot attribute the beneficial effect of the drug to an action solely in the CA1. Future studies should address whether GR blockade in the CA1 would have similar effects on HFD-induced OLM impairment.

Whereas the effects of RU in juvenile HFD-fed rats suggests that certain GR-dependent mechanisms are shared between LTP and memory, the effects of RU in CD animals are different. In CD juvenile animals the blockade of the GR does not affect OLM but results in inhibition of LTP, suggesting that normal activation of GR is required for intact LTP in CA1. This suggests that in some conditions, there exists a dissociation between LTP and memory as was previously reported using genetically-modified mouse or pharmacological tools (see for example^44–46^). Interestingly, Avital and colleagues^47^ showed that GR blockade led to a long-lasting increase of *in vivo* LTP in the dentate gyrus of adult rats. Future studies should address this intriguing opposite GR modulation of hippocampal synaptic plasticity in CA1 and dentate gyrus in adult male rats.

## Summary

We recently showed that 7 days of HFD exposure in juveniles impaired both LTP in prefrontal cortex and social recognition memory in an oxytocin-dependent manner^26^. The results of the present study add also hippocampal-dependent impairments following the acute consumption of HFD. In our previous studies, we showed differences in the emergence of the effects of HFD in the hippocampus. Specifically, we previously reported that 2–3 months of juvenile HFD exposure were sufficient to impair hippocampal-dependent memory^20,35^, whereas 1 month of HFD exposure starting at weaning only slightly attenuates long-term spatial memory^20^. However, in the present study we show that 7 days is enough and sufficient to induce hippocampal changes in juvenile and adult animals. This may suggest different waves/cycles that may induce an effect of HFD that is followed by a phase of adaptation, as recently shown in adult HFD-fed rats^15^. Future studies should address the temporal changes in hippocampal function following different durations of HFD exposure.

## Supplementary information

Supplementary data
