# Supplementary material for: Acute exposure to a high-fat diet in juvenile male rats disrupts hippocampal-dependent memory and plasticity through glucocorticoids
Source: Sci Rep. 2019 Aug 22;9:12270. doi: 10.1038/s41598-019-48800-2 (PMC6706405; doi:10.1038/s41598-019-48800-2)
Supplement: Supplementary file 1 — Supplementary data [file 41598_2019_48800_MOESM1_ESM.docx]

**Acute exposure to a high-fat diet in juvenile male rats disrupts hippocampal-dependent memory and plasticity through glucocorticoids**

Tala Khazen**^1^**, Ossama Abu Hatoum**^2*^**, Guillaume Ferreira**^3,4*^** and Mouna Maroun**^1*#^**

**^1^**Sagol Department of Neurobiology, Faculty of Natural Sciences, University of Haifa, Haifa 3498838, Israel.

**^2^** Department of Surgery B- HaEmek Medical Center, Faculty of Medicine, Technion: Israel Institute of Technology, Afula Israel

**^3^** INRA, Nutrition and Integrative Neurobiology, UMR1286, Bordeaux, France

**^4^** University of Bordeaux, Nutrition and Integrative Neurobiology, UMR 1286, Bordeaux, France

**^*^** Senior equal contribution

# Corresponding author

Mouna Maroun, Ph.D

Department of Neurobiology

University of Haifa, Haifa 31905, Israel

Email: [mouna.maroun@gmail.com](mailto:mouna.maroun@gmail.com)

**Supplemental data**

**Table S1.** Total exploration time in the different experiments and groups.

| **Experiment** | **Groups** | **Training exploration** | **Statistics** | **Test exploration** | **Statistics** |
| --- | --- | --- | --- | --- | --- |
|  |  |  |  |  |  |
| Long-term memory (LTM)  *Figure 1B* | jCD | 102.6 ± 15.7 | Diet, age or interaction:  F_(1,20)_<1 | 73.0 ± 14.2 | Diet, age or interaction:  F_(1,20)_<1 |
|  | jHFD | 109.0 ± 6.9 |  | 114.2 ± 5.0 |  |
|  | aCD | 90.8 ± 8.5 |  | 106.6 ± 15.1 |  |
|  | aHFD | 90.0 ± 12.3 |  | 107.9 ± 11.5 |  |
|  |  |  |  |  |  |
| Short-term memory  *Figure 1C* | jCD | 98.3 ± 10.5 | Diet, age or interaction:  F_(1,31)_<1 | 85.2 ± 10.0 | Diet, age or interaction:  F_(1,31)_<1 |
|  | jHFD | 87.9 ± 9.6 |  | 90.1 ± 10.0 |  |
|  | aCD | 106.0 ± 9.2 |  | 104.9 ± 11.6 |  |
|  | aHFD | 95.9 ± 9.5 |  | 96.1 ± 11.8 |  |
|  |  |  |  |  |  |
| LTM - RU  *Figure 4B* | jCD-Veh | 85.1± 15.7 | Diet: F_(1,32)_<1  Drug: F_(1,32)_=11.7, p<0.01  Interaction: F_(1,32)_=3.8, p=0.06 | 103.4 ± 10.8 | Diet: F_(1,32)_=6.7, p<0.05  Drug: F_(1,32)_=8.4, p<0.01  Interaction: F_(1,32)_=5.9, p<0.05 |
|  | jHFD-Veh | 115.7± 5.2 |  | 103.7 ± 5.2 |  |
|  | jCD-RU | 56.5 ± 7.7 |  | 65.6 ± 9.0 |  |
|  | jHFD-RU | 59.1 ± 10.1 |  | 67.1 ± 5.0 |  |
|  |  |  |  |  |  |
| LTM-RU delayed  *Figure S3* | jCD-Veh | 85.3 ± 15.7 | Diet, age or interaction:  F_(1,18)_<1 | 78.0 ± 12.3 | Diet or Age: F_(1,18)_<1  Interaction: F_(1,18)_=1.9, p=0.18 |
|  | jHFD-veh | 105 ± 9.2 |  | 95.2 ± 8.0 |  |
|  | jCD-RU | 81.0 ± 12.1 |  | 95.7 ± 7.8 |  |
|  | jHFD-RU | 82.7 ± 9.5 |  | 83.6 ± 9.9 |  |

Exploration times (s) of the different experimental groups in the training and the test sessions. Data are presented as means ± standard error of the mean.

**Fig. S1 juveniles:** Gel for the Western blot analysis of GR expression levels in CA1 in juvenile rats exposed for 7-9 days to control (CD) or high-fat diet (HFD).


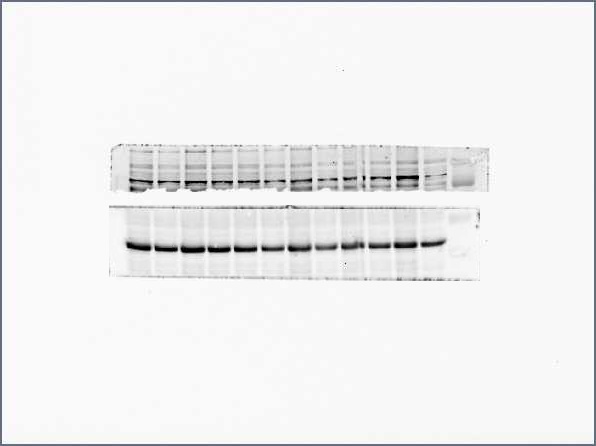


CD

CD

CD

CD

CD

CD

HFD

HFD

HFD

HFD

HFD

HFD

**Fig. S2 adults:** Gel for the Western blot analysis of GR expression levels in CA1 in adult rats exposed for 7-9 days to control (CD) or high-fat diet (HFD).


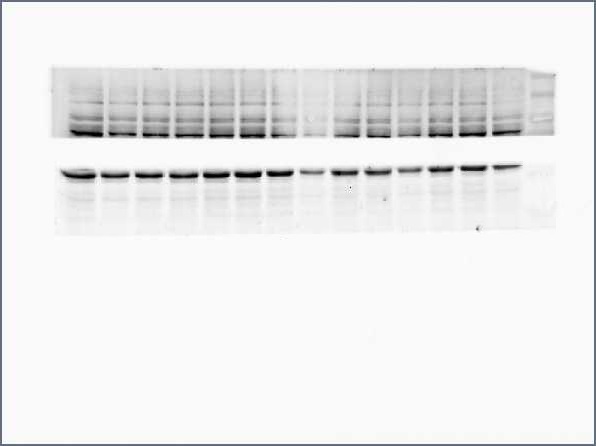


CD

CD

CD

CD

CD

CD

CD

HFD

HFD

HFD

HFD

HFD

HFD

HFD

**Fig. S3**: Effects of glucocorticoid receptors blockade 6 hours after training on juvenile HFD-induced impairments of object location memory. (A) Schematic representation of the experimental procedure. (B) HFD impaired long-term OLM in juveniles, and GR blockade with RU486 (RU) 6 hours after training did not rescue OLM in the HFD group [Diet effect: F(1,18)=85.1, ***P< 0.0001; Interaction Drug X Diet: F(1,18)<1].

**A**

18h


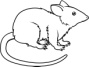

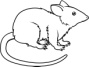


Vehicle

or RU

**Juvenile rats**

(*3 week-old*)

7-9 days of

CD or HFD

6h

**B**

**jCD**

***Veh-6h***

(n=6)

**jHFD**

***Veh-6h***

(n=5)

**jHFD**

***RU-6h***

(n=7)

**jCD**

***RU-6h***

(n=4)

Discrimination index

*******

**Long-term memory**

*******

**Fig. S4**: Acute exposure to HFD and GRs antagonist injection did not affect the distance travelled (A) or the anxiety index in the elevated plus maze.

**A**

**jCD**

***Veh***

(n=4)

**jHFD**

***Veh***

(n=4)

**jHFD**

***RU***

(n=7)

**jCD**

***RU***

(n=4)

**Total distance traveled (sec.)**

**B**

**Anxiety index**

**jCD**

***Veh***

(n=4)

**jHFD**

***Veh***

(n=4)

**jHFD**

***RU***

(n=7)

**jCD**

***RU***

(n=4)
